# Interobserver variability, detection rate, and lesion patterns of ^68^Ga-PSMA-11-PET/CT in early-stage biochemical recurrence of prostate cancer after radical prostatectomy

**DOI:** 10.1007/s00259-020-04718-w

**Published:** 2020-03-10

**Authors:** Jonathan Miksch, Dirk Bottke, Thomas Krohn, Reinhard Thamm, Detlef Bartkowiak, Christoph Solbach, Christian Bolenz, Meinrad Beer, Thomas Wiegel, Ambros J. Beer, Vikas Prasad

**Affiliations:** 1 Department of Nuclear Medicine, University Hospital of Ulm, Albert-Einstein-Allee 23, 89081 Ulm, Germany; 2 Department of Radiation Oncology, University Hospital of Ulm, Albert-Einstein-Allee 23, 89081 Ulm, Germany; 3 Department of Radiation Oncology, Esslingen Hospital, Hirschlandstraße 97, 73730 Esslingen, Germany; 4 Radiologie Aachen Land, Bahnhofstraße 17, 52146 Würselen, Germany; 5 Department of Urology, University Hospital of Ulm, Albert-Einstein-Allee 23, 89081 Ulm, Germany; 6 Department of Radiology, University Hospital of Ulm , Albert-Einstein-Allee 23, 89081 Ulm, Germany

**Keywords:** PET/CT, PSMA, Low PSA, Prostatectomy, Biochemical recurrence, Prostate cancer

## Abstract

**Purpose:**

^68^Ga-PSMA-11-PET/CT is increasingly used in early-stage biochemical recurrence of prostate cancer to detect potential lesions for an individualized radiotherapy concept. However, subtle findings especially concerning small local recurrences can still be challenging to interpret and are prone to variability between different readers. Thus, we analyzed interobserver variability, detection rate, and lesion patterns systematically in a homogeneous patient population with low-level biochemical recurrence.

**Methods:**

We analyzed ^68^Ga-PSMA-11-PET/CTs in 116 patients with status post-prostatectomy and PSA levels up to 0.6 ng/ml. None of them received ADT or radiotherapy beforehand. Images were interpreted and blinded by two nuclear medicine physicians (R1 and R2). Findings were rated using a 5-point scale concerning local recurrence, lymph nodes, bone lesions, and other findings (1: definitely benign, 2: probably benign, 3: equivocal, 4: probably malignant, 5: definitely malignant). In findings with substantial discrepancies of 2 or more categories and/or potentially leading to differences in further patient management, a consensus reading was done with a third reader (R3). Interobserver agreement was measured by Cohens Kappa analysis after sub-categorizing our classification system to benign (1 + 2), equivocal (3), and malignant (4 + 5). Time course of PSA levels after salvage treatment of patients rated as positive (4 + 5) was analyzed.

**Results:**

The overall detection rate (categories 4 and 5) was 50% (R1/R2, 49%/51%) and in the PSA subgroups 0–0.2 ng/ml, 0.21–0.3 ng/ml, and 0.31–0.6 ng/ml 24%/27%, 57%/57%, and 65%/68%, respectively. Local recurrence was the most common lesion manifestation followed by lymphatic and bone metastases. The overall agreement in the Cohens Kappa analysis was 0.74 between R1 and R2. For local, lymphatic, and bone sites, the agreement was 0.76, 0.73, and 0.58, respectively. PSA levels of PSMA PET/CT-positive patients after salvage treatment decreased in 75% (27/36) and increased in 25% (9/36). A decrease of PSA, although more frequent in patients with imaging suggesting only local tumor recurrence (86%, 18/21), was also observed in 67% (10/15) of patients with findings of metastatic disease.

**Conclusions:**

In a highly homogeneous group of prostate cancer patients with early-stage biochemical recurrence after radical prostatectomy, we could show that ^68^Ga-PSMA-11-PET/CT has a good detection rate of 50% which is in accordance with literature, with clinically relevant findings even in patients with PSA < 0.21 ng/ml. The interobserver variability is low, particularly concerning assessment of local recurrences and lymph nodes. Therefore, PSMA-PET/CT is a robust diagnostic modality in this patient group for therapy planning.

## Introduction

Prostate cancer (PCa) is the second most common malignancy in the male population worldwide [1]. Localized PCa is mainly treated with radical prostatectomy (RP), external beam radiotherapy (EBRT), or brachytherapy. However, between 27 and 53% of all patients undergoing radical prostatectomy or radiation therapy develop a rising PSA, termed as biochemical recurrence [2–4]. In patients with biochemical recurrence (BCR), studies have shown that ^68^Ga-PSMA-11-PET/CT is superior to F-18 or C-11 Cholin PET/CT in the detection of a correlate for rising PSA levels [5–8]. Even for BCR at low PSA levels (< 0.5 ng/ml), lesion detection for planning a locoregional therapy is possible [9–11]. A study investigating the detection efficacy of PSMA PET/CT in early-stage BCR revealed metastases even at very low PSA values down to 0.2 ng/ml [12]. The overall positive detection rate was 55% in patients with PSA 0.2–0.5 ng/ml and 74% in patients with PSA 0.5–1 ng/ml. In 80% and 70%, respectively, of these PSMA PET/CT-positive cases, tracer uptake occurred beyond the prostate bed, i.e., in lymph nodes, bones, or visceral organs [12]. Not surprisingly, it has been shown that PSMA PET/CT leads to 43% changes in staging and 59% in radiotherapy planning and thus is increasingly used for treatment planning of recurrent prostate cancer [13]. However, with therapeutic decisions based on ^68^Ga-PSMA-11-PET/CT results, it is of high relevance to understand and analyze the influence of interobserver variability and demonstrate the robustness of this still relatively novel method. This is especially true for low-level PSA values as the changes in management can be substantial and the findings in PET/CT might often be subtle. The impact of reader’s experience was analyzed by Fendler et al.: in a multicenter study, they reported a positive correlation between interobserver agreement and the readers’ experience and recommend further investigations, as pitfalls in image evaluation can occur independently from the level of knowledge [14]. Such pitfalls and equivocal uptakes could be seen in benign processes like Wegener’s granulomatosis, sarcoidosis, and Paget’s disease [15, 16] and also in malignancies such as multiple myeloma [17] and lung cancer [18]. These issues in PSMA imaging lead to the development of a 5-point scale standardized molecular imaging reporting and data systems (MI-RADS) by Werner et al. [19]. However, to the best of our knowledge, there are no data on interobserver variability of ^68^Ga-PSMA-11-PET/CT in the clinically relevant patient population with low-level biochemical recurrence after RP.

Thus, in this study, we evaluated the performance and interreader variability of ^68^Ga-PSMA-11-PET/CT in a homogeneous patient population of therapy-naive PCa patients with BCR after RP with PSA < 0.6 ng/ml.

## Material and methods

### Patients

All patients included gave written and informed consent for the ^68^Ga-PSMA-11-PET/CT examination and in the retrospective data analysis, and study protocol was approved by the local ethics committee of the University of Ulm (152/19-Fst/bal.)

### Study design

We performed a retrospective analysis on 1026 histologically confirmed prostate cancer patients who underwent a ^68^Ga-PSMA-11-PET/CT during the period from January 2013 until May 2016 in the department of nuclear medicine at the University Hospital Ulm. One hundred sixteen patients with status post-prostatectomy and PSA recurrence up to 0.6 ng/ml and no prior ADT, radiotherapy, or chemotherapy were elected for this analysis. PSA values were not older than 6 weeks prior to the PSMA PET/CT. In Table 1, patients’ characteristics are summarized. ^68^Ga-PSMA-11-PET/CT were evaluated by two readers (R1 and R2) to analyze interobserver variability and for consensus finding a third reader (R3) reviewed critical cases. For follow-up, we analyzed the time course of PSA levels within 1 year after salvage treatment of patients rated as PSMA-PET/CT-positive by R1. A decrease of ≥ 50% after salvage treatment was considered a sufficient PSA response. A rising PSA value or a PSA decrease of < 50% was defined as an insufficient PSA response.

**Table 1:** Patient and tumor characteristics

Characteristics (*n* = 116)	Mean, absolute number, and/or percentage value
Age	67.6 (48–84)
Clinical information	
Initial PSA value	10.6 (1.93–37)
PSA value before PET/CT	0.26 (0.02–0.55)
Tumor stage (TNM) (*n* = 114)	
Local tumor	114 (100%)
*N* positive	11 (10%)
Gleason score (*n* = 111)	
≤ 6	20 (18%)
7	64 (58%)
≥ 8	27 (24%)

### Positron emission tomography imaging

The precursor PSMA-HBED-CC (PSMA-11) was purchased from ABX GmbH (Radeberg, Germany). The radiopharmaceutical ^68^Ga-PSMA-HBED-CC ([^68^Ga]GaPSMA-11) was produced as recently published [20, 21]. For radiolabeling, a 50 mCi (1850 MBq) ^68^Ge/^68^Ga radionuclide generator was used (iThemba LABS, South Africa).

PET/CT image acquisition was performed with a 40-slice CT with two overlapping X-ray beams and a 21.8 cm axial field of view PET detector Biograph mCT (40)S (Siemens Biograph mCT(40)S, Siemens Healthineers, Erlangen, Germany) 64.4 ± 12.2 min after intravenous application of 162.7 ± 22.3 MBq ^68^Ga-PSMA-11. Fifteen to twenty milligrams of furosemide was injected i.v. to enhance diuresis. First, a diagnostic CT scan was performed in the portal venous phase 80 s after intravenous injection of contrast agent (80 to 120 ml Ultravist 370, Bayer Schering Pharma, Berlin, Germany) in 105 patients (91%) and without contrast agent in 11 patients (9%). CT scans were done using attenuation-based online modulation of tube current (CARE Dose) with quality reference tube current setting (reference mAs) of 210 mAs, 120 kV, 0.5 s per rotation, 16 × 1.2-mm collimation followed by the PET scan from the mid-thighs to the vertex in 5 to 8 bed positions. A separate low-dose CT of the chest in deep inspiration was performed in all patients (quality reference tube current setting of 25 mAs, 120 kV). All patients received oral contrast (300 mg Telebrix). All PET scans were acquired in 3D mode with an acquisition time of 3 min per bed position in time of flight technique.

### Image analysis

**Fig. 1:**
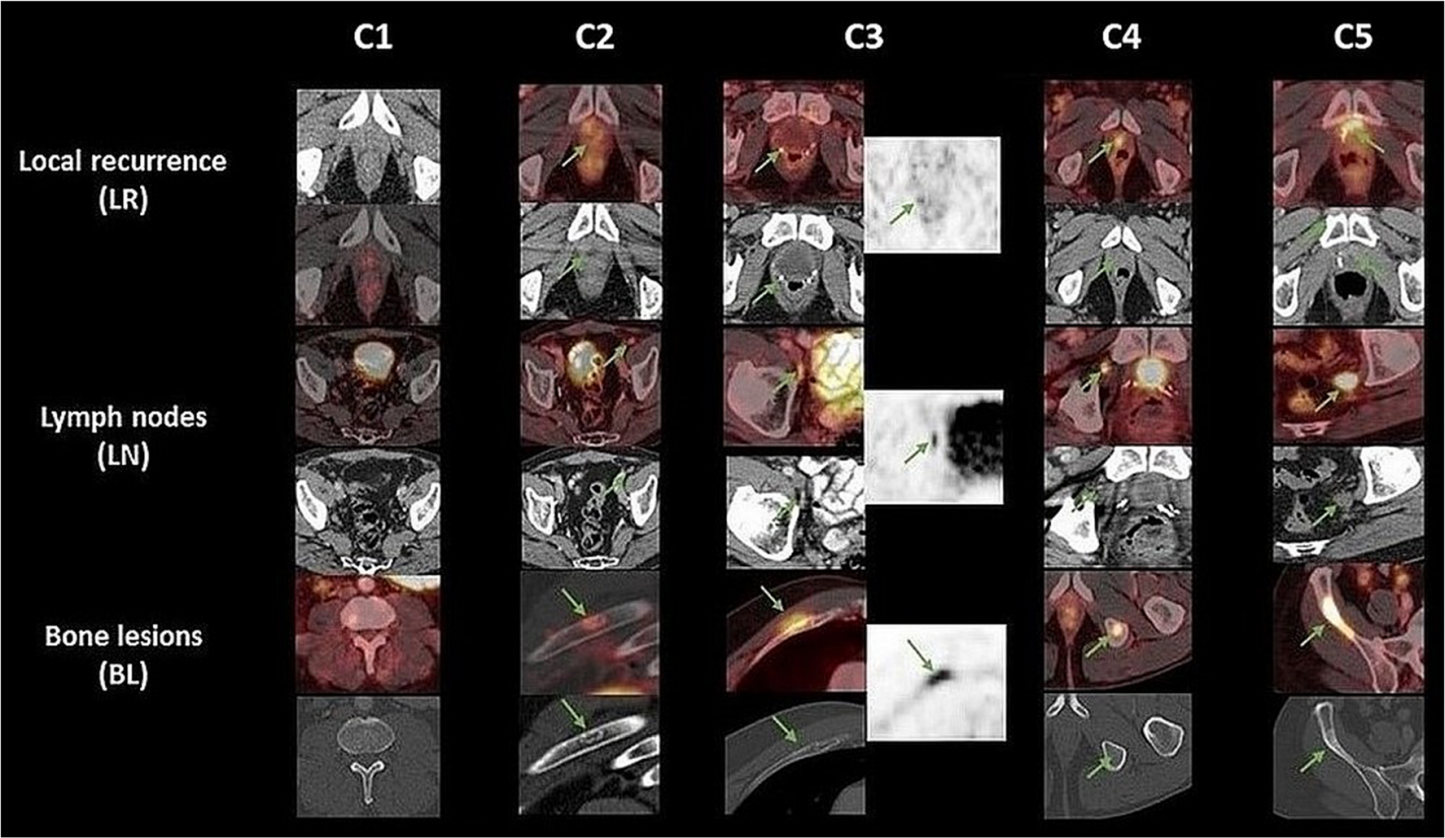
Fused PET/CT and CT image examples of the categories 1–5 (C1–C5) for each lesion type. Green arrows indicate the findings. C1 definitive benign: Local recurrence (LR): no suspicious findings; Lymph nodes (LN): small lymph nodes without uptake; Bone lesions (BL): no suspicious findings. C2 probably benign: LR: low diffuse uptake, most likely reactive, no CT correlate (e.g. shortly after RRP); LN: low to moderate uptake, typical site of inflammatory LN, like groin; BL: moderate uptake but typical CT patterns of benign lesions. C3 equivocal; LR: focal but low uptake on only 1-2 slices, no CT correlate, DD scatter; LN: moderate to intense uptake, but in area with high rate of inflammatory LN; BL: moderate to intense uptake, no CT correlate, but in area prone to false positives, e.g. ribs; C4 probably malignant: LR: focal moderate to intense uptake, typical site of LR, no CT correlate; LN: intense uptake, typical site of LN metastases, not enlarged in CT; BL: intense focal uptake, typical site of bone metastases, no CT correlate; C5 definitive malignant: LR: focal intense uptake, typical site of LR, with CT correlate; LN: intense uptake, typical site of LN metastases, enlarged in CT; BL: intense focal uptake, typical site of bone metastases, with typical CT correlate. For equivocal lesions (C3), PET-only images are also displayed. Note that these findings are the most challenging. Here often further work-up is necessary, e.g., follow-up imaging, additional imaging like MRI, or even biopsy

Images were evaluated by an experienced board-certified radiologist and nuclear medicine physician (R1) and an experienced board-certified nuclear medicine physician (R2). The readers were given the information of a biochemical recurrence with PSA range of 0–0.6 ng/ml and that all patients were status post-prostatectomy. In case of discrepant classifications of R1 and R2, a third board-certified nuclear medicine physician (R3) assessed the cases for consensus finding. All three readers had more than 10-year experience in hybrid image evaluation with over 5-year experience in reading PSMA PET scans. Lesions were classified as local recurrences, lymphatic metastases, bone metastases, or other lesions and were evaluated on a 5-point scoring system with 1: definitely benign, 2: probably benign, 3: equivocal, 4: probably malignant, and 5: definitely malignant. For definition of the criteria for each category and representative image examples, see also Fig. 1. Similar scoring systems were also used by other groups in recent studies [15, 22, 23]. For the analysis of detection rates, categories 4 and 5 were considered “positive for malignancy” and 1–3 as “negative.” In patients with multiple lesions, the lesion with the highest score determined the overall score for the patient.

### Statistics

Descriptive statistics are mentioned as frequency, mean, median, standard deviation, and range wherever necessary. Interobserver variability was assessed by Cohen’s kappa analysis [24]. Values of 0–0.20 define poor, 0.21–0.40 fair, 0.41–0.60 moderate, 0.61–0.80 substantial, and 0.81 to 1.0 a nearly perfect agreement. For this analysis, the scores of 1 and 2 were rated as “benign,” 3 as “equivocal,” and 4 and 5 as “malignant.” The statistical analysis was performed on IBM SPSS software version 24.

## Results

### Detection rate

After consensus reading, the overall detection rate (lesions of categories 4 and 5) in our patient population was 50%. The detection rate increased with rising PSA levels with 27% below 0.2 ng/ml, 55% in the range from 0.21 to 0.3 ng/ml and 68% in the highest range from 0.31 to 0.6 ng/ml. There was no substantial correlation of positive findings with the Gleason score, with detection rates of 45%, 47%, and 59% for Gleason score subgroups of ≤ 6, 7, and ≥ 8.

**Fig. 2:**
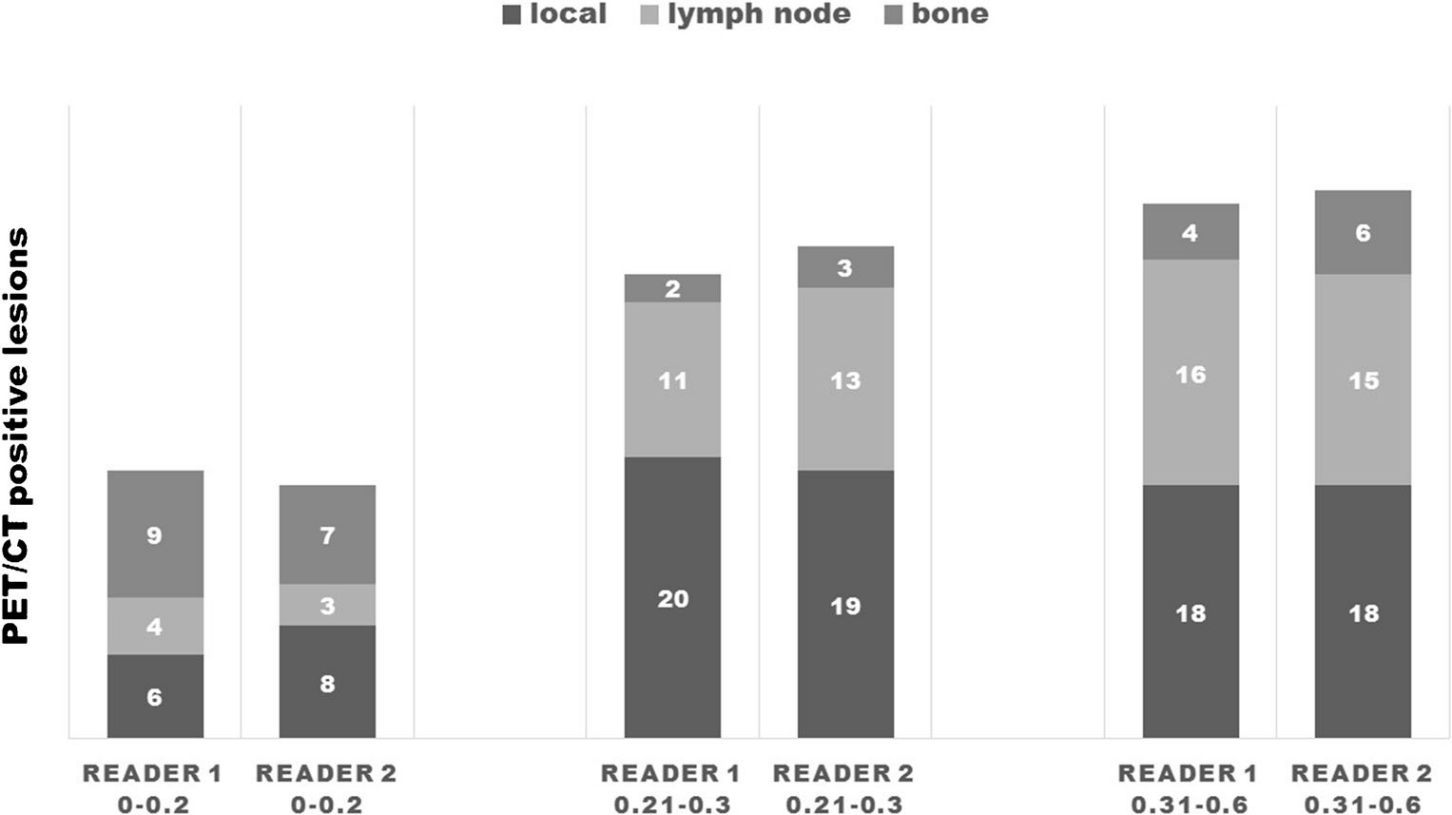
Distribution of the positive lesions in PSA subgroups. In the lowest PSA subgroup, 0–0.2 ng/ml surprisingly bone metastases are the most frequent tumor manifestations. This is in part due to one patient with several bone lesions. In the subgroups with higher PSA, local recurrences occurred most frequently, followed by lymphatic and bone lesions.

Local recurrence was the most common lesion manifestation followed by lymphatic and bone metastases. The frequency of lymph node metastases and bone metastases increased with rising PSA levels (see also Fig. 2). Distant metastases were also noted even in the subgroup with the lowest PSA level. However, the high number of bone metastases in this subgroup is potentially biased by one patient with multiple suspicious osseous foci.

### Interobserver variability

The overall agreement was 0.74 between R1 and R2 (Cohen’s kappa analysis). For local, lymphatic and bone sites, the agreement was 0.76, 0.73, and 0.58 respectively. In total, 90 lesions in 57 patients were rated as malignant (scores 4 or 5) by R1 and 92 lesions in 59 patients by R2. On a per patient basis, R1/R2 rated 49%/51% of the patients as positive (scores 4 or 5). Detection rates (patient-based) by R1 and R2 increased with rising PSA, 24% vs. 27% (PSA 0–0.2 ng/ml), 57% vs. 57% (PSA 0.21–0.3 ng/ml), and 65% vs. 68% (PSA 0.31–0.6 ng/ml). Detection rates according to the Gleason score were 45%/45% (GS ≤ 6), 47%/48% (GS 7), and 59%/59% (GS ≥ 8). The distribution of positive findings among anatomic regions was well matched between R1 and R2: 44 vs. 45 local recurrences, 31 vs. 31 lymphatic lesions, and 15 vs. 16 bone metastases, respectively.

**Fig. 3:**
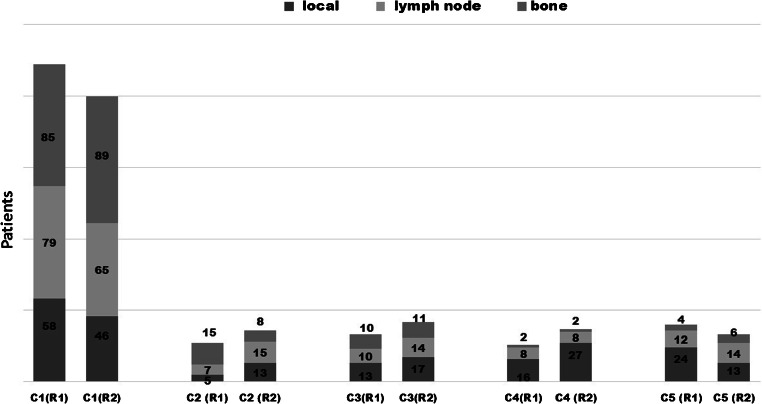
Distribution of the 116 patients ratings in the five categories (C1–C5) and the three lesion sites (local, lymph node, bone) by R1 and R2. The highest rated lesion determined the score for each lesion site resulting in 348 score values by R1 and R2

**Fig. 4:**
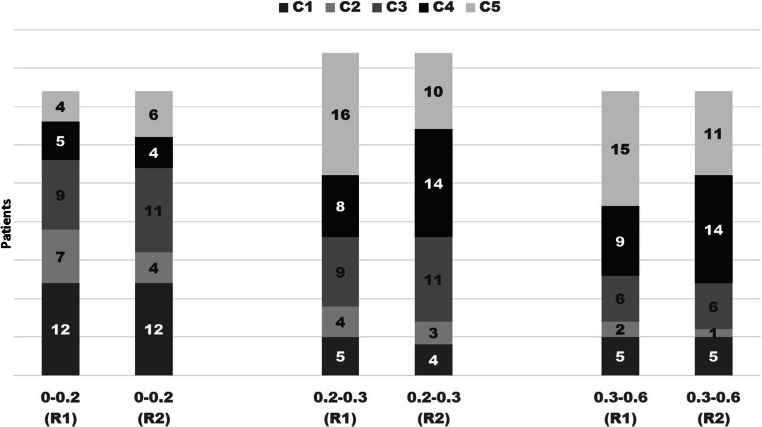
PSA subgroups and categories by R1 and R2. Note that the patients’ benign and equivocal ratings are well matched between the readers, while in the PSMA-PET/CT positive scores in the higher PSA subgroups more discordancies occurred

The ratings of both readers for different lesion subtypes were comparable and are summarized in detail in Fig. 3. The levels of confidence were highest in the subgroup with PSA 0.2–0.3 ng/ml, for details, see Fig. 4. Clinically relevant discrepancies between both readers (i.e., either a difference of 2 categories or more, or a change from the “positive group” with 4–5 to the “negative group” with 1–3 or vice versa) were highest (patient-based) for local recurrences in 14/116 (12%) followed by lymph nodes in 12/116 (10%) and bone lesions in 8/116 (7%). Fifty-three percent (18/34) of discrepancies were in the “negative group,” which means that discrepancies were between rating 1 and 3, which however usually is of lesser clinical relevance. Table 2 summarizes the potential reasons of such discrepancies.

**Table 2:** Probable causes of discrepancies between R1 and R2 enlarge nodes with low

Lesion localization	Causes of discrepancies
Former prostatic fossa and seminal vesicle	-Post surgical changes (e.g., clips) -High activity in bladder -Discrepancy between PET signal and CT patterns
Lymphatic system	-Enlarge nodes with low/moderate PSMA expression -Suspect findings in atypical locations (e.g., inguinal, axillar, periclavicular lymph nodes) -Differentiation between reactive and pathologic lymph nodes -Second malignancy (e.g., lymphoma) -Benign lymphoproliferative diseases (e.g., sarcoidosis)
Bone site	-Fibro-osseous lesions -Special case in low PSA levels: low probability of bone metastases -Morphologic suspicious lesions with no/low PSMA expression -Suspicious uptake near fracture/degeneration(e.g., ribs) -Lesions close to joints

### Follow-up

**Fig. 5:**
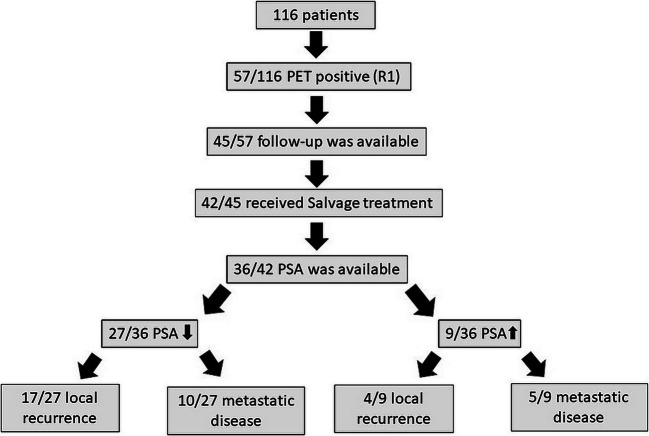
Follow-up of patients evaluated as PSMA-PET/CT-positive. After salvage treatment, the majority of patients showed a sufficient PSA response

The time course of PSA values of patients rated as positive by R1 after salvage treatment is outlined in Fig. 5. In this group of patients, 75% (27/36) showed a sufficient PSA response, whereas in 25% (9/36), there was no sufficient PSA response. Ninety-three percent (25/27) of patients with a sufficient PSA response presented values below the detection limit. Eighty-one percent (17/21) of patients rated as LR only showed a sufficient PSA response. In patients rated as having metastatic disease, 67% (10/15) showed a sufficient PSA response.

## Discussion

In this study, we could show in a well characterized and highly homogeneous population of PCa patients with BCR after RP that PSMA PET/CT findings are robust and reliable and offer an excellent detection rate even in patients with low PSA values < 0.6 ng/ml. Interreader agreement is good especially concerning assessment of local recurrences and lymph node metastases.

### Detection rates

Our detection rates are generally in line with most published reports on comparable patient populations with low-level BCR after RP, like the data from van Leeuwen et al. with 54% and Maurer et al. with 54% [25, 26]. Also, in more heterogeneous patient populations, similar results were reported [8–11]. Slightly discrepant results with lower detection rates are reported by Farolfi et al. who analyzed 119 patients with status post-prostatectomy and PSA levels between 0.2 and 0.5 ng/ml with a rather low detection rate of 34% [27]. This discrepancy can be explained by differences in the patient population: while in our study only patients who did not receive any kind of therapy post-RP were included, in the study by Farolfi et al., radiation therapy and ADT were not clearly excluded.

In our patients, we observed no substantial correlation of detection rates and higher Gleason scores. However, as Gleason score 7 was diagnosed in more than half of our collective, this result has to be interpreted with caution. A positive influence of the Gleason score on the PSMA expression has been seen in preclinical trials as well as in studies by Eiber et al. and Marchal et al. [9, 28]. On the contrary, in clinical trials by Afshar-Oromieh et al., no such positive correlation was found [23, 29]. Of special clinical relevance is the fact that even in the subgroup with the lowest PSA levels, distant metastases could be identified, which has a substantial impact on clinical management.

As we did not have histopathological validation for the majority of imaging findings, we analyzed the time course of PSA values of patients rated positive by R1 after salvage treatment as a surrogate parameter to validate our results. The majority of patients showed a sufficient PSA response (75%), which suggests that our findings were correct in most cases. Results were even better for patients where imaging suggested local recurrence as the only finding, with 81% PSA response after salvage treatment. Only a minority of patients showed no sufficient PSA response despite salvage treatment (25%), which means that we missed malignant lesions in this group. However, in this group, most patients had findings of metastatic disease (56%), which suggests that PSMA imaging is able to define two risk groups even at very low levels of PSA recurrence after prostatectomy: One with PSMA-PET/CT findings of only local tumor recurrence and excellent response to salvage treatment and a second group with findings of metastatic disease and lower response rates. However, even in the group of patients with findings suggestive of metastatic disease, 67% had a sufficient PSA response after salvage treatment. This is a promising result keeping in mind that formerly without PSMA-PET/CT guided treatment many lesions would have been missed in this group.

### Interobserver variability

For investigating the interobserver variability, a homogeneous set of readers and strict definition of criteria for lesion classification are mandatory. In our study, we optimized these prerequisites by choosing readers with similar background and experience in reading PSMA PET scans. In addition, we defined strict criteria for classification of lesions for local recurrences, lymph nodes, and bone lesions. In the literature, different strategies have been used to assess the interreader variability. Afshar-Oromieh et al. used two different protocols: in the first protocol, first two nuclear medicine physicians evaluated the data separately and afterwards together [29], and another protocol where two nuclear medicine physicians together with an assistant radiology physician supervised by a board-certified radiology physician evaluated the image series and a board-certified nuclear medicine physician reevaluated the patients PSMA PET/CT [10]. In two separate studies, authors have evaluated the interreader variability of a nuclear medicine physician and a radiologist and compared the results with consensus finding [9, 30]. A detailed description of the differences in reading between the physicians is not documented in the studies cited here.

Recently, various approaches have been reported in the literature concerning standardized evaluation systems for image evaluation and detection rates of PSMA PET/CTs, similar to the system we used in this study. Eiber et al. tried to objectify PSMA uptakes using the different tissue-specific physiological biodistribution of the PSMA agent. In their report system PROMISE, scores of 0, 1, 2, and 3 are given for lesions with no, low, average, and high tracer uptakes. The uptake in the vessels, liver, and spleen and the high activities of the parotid are used for comparison and to assess the likelihood for a malignant process [31]. A score value of 2 and 3 is considered to be highly suggestive for prostate cancer. Werner et al. also used a 5-score system to differentiate between benign, equivocal, and malignant tracer uptakes in PSMA imaging [19]. In our study, we integrated both PET and CT information for lesion localization as well as for characterization. This integration of both PET and CT patterns and localization of lesions is a prerequisite for a reliable interpretation of findings in PET/CT. The approach proposed by Werner et al. is somewhat similar to the one used in our study.

The substantial interobserver agreement of 0.74 in the current study is in line with data from the literature. Statistical analyses of the interobserver agreement were performed using the Cohen kappa as only two readers were involved in our survey. Krippendorff’s alpha is suitable for interreader differences of more than 2 readers and can be compared directly with the Cohen kappa. Fendler et al. did also describe a high consensus with [^68^Ga]PSMA-11 in an experienced reader group. However, their patient population also included patients with high PSA levels and patients with primary diagnosis as well as confirmed metastatic disease and thus was not as homogeneous as in our study [14]. Fanti et al. investigated the interreader agreement in a multicenter study resulting in the Krippendorff alpha scores of 0.68 for local site, 0.76 for loco regional lymph nodes, and 0.79 for bone findings [32]. However, in their study, a biochemical recurrence situation was the only inclusion criteria, contrary to our study. Moreover, the median PSA of 0.24 ng/ml (range 0.02–0.6 ng/ml) in our patient population was also lower compared with other studies focusing on interreader agreement in PSMA imaging. This could be one explanation concerning the lower interreader agreement of 0.58 for bone metastases as the probability and number of metastases in bone is much lower in patients with early-stage biochemical recurrences. In other studies, either lymphatic or bone lesions were the leading manifestations of prostate cancer seen in the PSMA scan.

### Limitations

Our analysis shares the general limitation of studies of imaging in recurrent PCa patients, i.e., due to practical and ethical issues, histopathological confirmation of the findings is missing. However, this might be of lesser importance for an analysis of interobserver variability, which was the focus of our study. Moreover, we analyzed the time course of PSA values after salvage treatment in patients rated as positive as “best valuable comparator” (BVC), which is an established method for follow-up of imaging findings [33].

## Conclusion

In a highly homogeneous group of prostate cancer patients with biochemical recurrence after prostatectomy, we could show that ^68^Ga-PSMA-11-PET/CT has very good detection rates even at PSA < 0.6 ng/ml. Low interreader variability between experienced readers, specifically for local recurrence and lymphatic disease, suggests that PSMA PET/CT findings are robust and reliable for therapy planning.

## References

[1] Latest global cancer data: Cancer burden rises to 18.1 million new cases and 9.6 million cancer deaths in 2018. IARC, WHO. 2018 https://www.who.int/cancer/PRGlobocanFinal.pdf. Accessed 25/08/19.

[2] Cetin K, Beebe-Dimmer JL, Fryzek JP, Markus R, Carducci MA (2010). Recent time trends in the epidemiology of stage IV prostate cancer in the United States: analysis of data from the Surveillance, Epidemiology, and End Results Program. Urology..

[3] Mottet N, Bellmunt J, Representative EBP, Bolla M, Bourke L, Vice-chair PC (2017). Prostate cancer EAU guidelines 2017. Eur Urol.

[4] SEER Cancer Stat Facts: Prostate cancer. National Cancer Institute. Bethesda, MD. https://seer.cancer.gov/statfacts/html/prost.html. Accessed 25/7/19.

[5] Krause BJ, Souvatzoglou M, Tuncel M, Herrmann K, Buck AK, Praus C (2008). The detection rate of [11C]choline-PET/CT depends on the serum PSA-value in patients with biochemical recurrence of prostate cancer. Eur J Nucl Med Mol Imaging.

[6] Beer AJ, Eiber M, Souvatzoglou M, Schwaiger M, Krause BJ (2011). Radionuclide and hybrid imaging of recurrent prostate cancer. Lancet Oncol.

[7] Giesel FL, Fiedler H, Stefanova M, Sterzing F, Rius M, Kopka K (2015). PSMA PET/CT with Glu-urea-Lys-(Ahx)-[68Ga(HBED-CC)] versus 3D CT volumetric lymph node assessment in recurrent prostate cancer. Eur J Nucl Med Mol Imaging.

[8] Perera M, Papa N, Christidis D, Wetherell D, Hofman MS, Murphy DG (2016). Sensitivity, specificity, and predictors of positive 68Ga-prostate-specific membrane antigen positron emission tomography in advanced prostate Cancer: a systematic review and meta-analysis. Eur Urol.

[9] Eiber M, Maurer T, Souvatzoglou M, Beer AJ, Ruffani A, Haller B (2015). Evaluation of hybrid ^68^Ga-PSMA ligand PET/CT in 248 patients with biochemical recurrence after radical prostatectomy. J Nucl Med.

[10] Afshar-Oromieh A, Holland-Letz T, Giesel FL, Kratochwil C, Mier W, Haufe S (2017). Diagnostic performance of 68Ga-PSMA-11 (HBED-CC) PET/CT in patients with recurrent prostate cancer: evaluation in 1007 patients. Eur J Nucl Med Mol Imaging.

[11] Giesel FL, Knorr K, Spohn F, Will L, Maurer T, Flechsig P (2019). Detection efficacy of [18F]PSMA-1007 PET/CT in 251 patients with biochemical recurrence after radical prostatectomy. J Nucl Med.

[12] Rauscher I, Düwel C, Haller B, Rischpler C, Heck MM, Gschwend JE (2018). Efficacy, predictive factors, and prediction nomograms for 68Ga-labeled prostate-specific membrane antigen-ligand positron-emission tomography/computed tomography in early biochemical recurrent prostate cancer after radical prostatectomy. Eur Urol.

[13] Habl G, Sauter K, Schiller K, Dewes S, Maurer T, Eiber M (2017). 68 Ga-PSMA-PET for radiation treatment planning in prostate cancer recurrences after surgery: individualized medicine or new standard in salvage treatment. Prostate..

[14] Fendler WP, Calais J, Allen-Auerbach M, Bluemel C, Eberhardt N, Emmett L (2017). 68Ga-PSMA-11 PET/CT interobserver agreement for prostate cancer assessments: an international multicenter prospective study. J Nucl Med.

[15] Sheikhbahaei S, Afshar-Oromieh A, Eiber M, Solnes LB, Javadi MS, Ross AE (2017). Pearls and pitfalls in clinical interpretation of prostate-specific membrane antigen (PSMA)-targeted PET imaging. Eur J Nucl Med Mol Imaging.

[16] Prasad V, Steffen IG, Diederichs G, Makowski MR, Wust P, Brenner W (2016). Biodistribution of [(68)Ga]PSMA-HBED-CC in patients with prostate cancer: characterization of uptake in normal organs and tumour lesions. Mol Imaging Biol.

[17] Rauscher I, Maurer T, Steiger K, Schwaiger M, Eiber M (2017). Image of the month: multifocal 68Ga prostate-specific membrane antigen ligand uptake in the skeleton in a man with both prostate cancer and multiple myeloma. Clin Nucl Med.

[18] Pyka T, Weirich G, Einspieler I, Maurer T, Theisen J, Hatzichristodoulou G (2016). 68Ga-PSMA-HBED-CC PET for differential diagnosis of suggestive lung lesions in patients with prostate cancer. J Nucl Med.

[19] Werner RA, Bundschuh RA, Bundschuh L, Javadi MS, Higuchi T, Weich A (2018). Molecular imaging reporting and data systems (MI-RADS): a generalizable framework for targeted radiotracers with theranostic implications. Ann Nucl Med.

[20] Eder M, Schäfer M, Bauder-Wüst U, Hull W-E, Wängler C, Mier W (2012). 68Ga-complex lipophilicity and the targeting property of a urea-based PSMA inhibitor for PET imaging. Bioconjug Chem.

[21] Schäfer M, Bauder-Wüst U, Leotta K, Zoller F, Mier W, Haberkorn U (2012). A dimerized urea-based inhibitor of the prostate-specific membrane antigen for 68Ga-PET imaging of prostate cancer. EJNMMI Res.

[22] Rauscher I, Maurer T, Fendler WP, Sommer WH, Schwaiger M, Eiber M (2016). (68)Ga-PSMA ligand PET/CT in patients with prostate cancer: how we review and report. Cancer Imaging.

[23] Afshar-Oromieh A, Malcher A, Eder M, Eisenhut M, Linhart HG, Hadaschik BA (2013). PET imaging with a [68Ga]gallium-labelled PSMA ligand for the diagnosis of prostate cancer: biodistribution in humans and first evaluation of tumour lesions. Eur J Nucl Med Mol Imaging.

[24] Landis JR, Koch GG (1977). The measurement of observer agreement for categorical data. Biometrics..

[25] van Leeuwen PJ, Stricker P, Hruby G, Kneebone A, Ting F, Thompson B (2016). (68) Ga-PSMA has a high detection rate of prostate cancer recurrence outside the prostatic fossa in patients being considered for salvage radiation treatment. BJU Int.

[26] Maurer T, Gschwend J, Wester H-J, Souvatzoglou M, Beer A, Holzapfel K, et al. PET imaging with 68 gallium-labelled ligand of prostate-specific membrane antigen ( 68 Ga-HBED-PSMA) for staging of biochemical recurrent prostate cancer after radical prostatectomy. J Clin Oncol. 2015;33:5023–3.

[27] Farolfi A, Ceci F, Castellucci P, Graziani T, Siepe G, Lambertini A (2019). 68Ga-PSMA-11 PET/CT in prostate cancer patients with biochemical recurrence after radical prostatectomy and PSA <0.5 ng/ml. Efficacy and impact on treatment strategy. Eur J Nucl Med Mol Imaging.

[28] Marchal C, Redondo M, Padilla M, Caballero J, Rodrigo I, García J (2004). Expression of prostate specific membrane antigen (PSMA) in prostatic adenocarcinoma and prostatic intraepithelial neoplasia. Histol Histopathol.

[29] Afshar-Oromieh A, Avtzi E, Giesel FL, Holland-Letz T, Linhart HG, Eder M (2015). The diagnostic value of PET/CT imaging with the (68)Ga-labelled PSMA ligand HBED-CC in the diagnosis of recurrent prostate cancer. Eur J Nucl Med Mol Imaging.

[30] Einspieler I, Rauscher I, Düwel C, Krönke M, Rischpler C, Habl G (2017). Detection efficacy of hybrid 68Ga-PSMA ligand PET/CT in prostate cancer patients with biochemical recurrence after primary radiation therapy defined by Phoenix Criteria. J Nucl Med.

[31] Eiber M, Herrmann K, Calais J, Hadaschik B, Giesel FL, Hartenbach M (2018). Prostate cancer molecular imaging standardized evaluation (PROMISE): proposed miTNM classification for the interpretation of PSMA-ligand PET/CT. J Nucl Med.

[32] Fanti S, Minozzi S, Morigi JJ, Giesel F, Ceci F, Uprimny C (2017). Development of standardized image interpretation for 68Ga-PSMA PET/CT to detect prostate cancer recurrent lesions. Eur J Nucl Med Mol Imaging.

[33] Janssen JC, Meißner S, Woythal N, Prasad V, Brenner W, Diederichs G (2018). Comparison of hybrid 68Ga-PSMA-PET/CT and 99mTc-DPD-SPECT/CT for the detection of bone metastases in prostate cancer patients: additional value of morphologic information from low dose CT. Eur Radiol.

